# Growing-rod implantation improves nutrition status of early-onset scoliosis patients: a case series study of minimum 3-year follow-up

**DOI:** 10.1186/s12893-021-01120-7

**Published:** 2021-03-01

**Authors:** Xingye Li, Zheng Li, Youxi Lin, Haining Tan, Chong Chen, Jianxiong Shen

**Affiliations:** 1 Department of Orthopaedics, Peking Union Medical College Hospital, Chinese Academy of Medical Sciences, Peking Union Medical College, Shuaifuyuan #1, Dongcheng District, Beijing, 100730 China; 2 Department of Orthopaedics, Peking University Fourth Clinical Medical College, Beijing Jishuitan Hospital, Beijing, 100035 China; 3 Department of Orthopaedics, Guangdong Provincial People’s Hospital, Guangzhou, 510080 China

**Keywords:** Early-onset scoliosis, Malnutrition, Growing-rod

## Abstract

**Background:**

Early onset scoliosis (EOS) may cause malnutrition in affected patients. Growing-rod treatment has been an effective protocol for treating EOS. The objective of this study is to demonstrate whether growing-rod treatment improves nutritional status of EOS patients.

**Methods:**

Fifty-two EOS patients who had dual growing-rod surgery was enrolled. The minimum follow-up was 3-years. Their body weights were normalized based on the data of two National Population Census of China. Z-scores were used to indicate the standard deviation from the median body weight-for-age.

**Results:**

The median follow-up time was 6 years. Preoperatively, the prevalence of malnutrition (Z < − 2) was 21.2%, and reduced to 9.6% at the end of the follow-up. Preoperatively, the average Z-score was − 0.94, and it increased to − 0.65 at the latest follow-up (p < 0.05). Patients with preoperative Z-score below − 1 had more significant increase of Z-scores (− 2.15 vs − 1.26, p < 0.001). A significant negative correlation between the change of Z-score and the preoperative Z-score (correlation coefficient − 0.65, p < 0.001).

**Conclusions:**

The growing rod surgery and lengthening procedures significantly improves the nutrition status of EOS patients. The body weight gains are more significant in patients with lower body weights.

## Introduction

Early-onset scoliosis (EOS) compromises chest-wall movement [1–3] and may lead to respiratory disorders and pulmonary insufficiency [4–7]. In diseased children, the energy consumption of the work of breathing increases, that leads to additional basal energy consumption [8]. On the other hand, nutritional intake activities may be impaired in scoliotic patients [9]. As a result, children with EOS may develop malnutrition.

EOS patients were treated by a series of growth-friendly techniques. Growing-rod technique was proved to reduce the angle of the curves, lengthen T1–T12 distance, and promote lung development in EOS patients [10, 11]. Thus, growing-rod technique may have role in improving nutritional status of EOS patients. Myung et al. found increase in postoperative weight percentile at minimum 2-years follow-up in EOS patients with growing-rod surgery [12]. Harris et al. found growing-rod resulted increase in weight percentile for underweight patients [13]. Another growth friendly technique including vertical expandable prosthetic titanium rib (VEPTR) was also proved to increase weight for EOS patients [14]. However, previous studies did not reveal the age and gender adjusted body-weight position change of the patient among the large population.

The purpose of our study was to show whether growing-rod technique improves nutritional status of EOS patients based on data of National Population Censuses of China.

## Methods

This study collected EOS cases in Peking Union Medical College Hospital from 2000 to 2020. The patient was included if he/she (1) had a posterior growing-rod implantation and lengthening procedures; (2) had a minimum follow-up of 36 months. The patient was excluded if he/she (1) has congenital heart disease; (2) has disease in alimentary tract. Medical records of involved patients were reviewed. Their age, body weight, height, Cobb angle at initial surgery, as well body weight and age at each follow up were recorded. Personal information of enrolled patients was kept confidential, and written informed consent was obtained from the patient and their legal guardians.

The weight-for-age position of the patients among the general Chinese population was determined by the growth reference curve, which was published by Li et al. in 2013 [15]. The reference curve was constructed based on 94,302 school children derived from two cross-sectional National Population Censuses in China using Cole’s LMS method. At each age from 0 to 18 years old, the skewness (*L*), the median (*M*) and the coefficient of variation (*S*) were given in the above literature. The weight-for-age can be normalized into Gaussian distribution:

$$Body\,weight\, = \,{\text{M}}\left( {1\, + \,{\text{L}}\, \times \,{\text{S}}\, \times \,{\text{Z}}} \right)^{1/L}$$

where *Z* is the Z-score, representing the standard deviation (SD) from the median body weight of the general population at a certain age. Z can be deducted from above equation:

$$Z=\frac{\left(\frac{Body\;weight}M\right)^L-1}{L\times S}$$

According to The World Health Organization Global Database on Child Growth and Malnutrition, Z-score below − 2 is commonly considered as the cut-off of malnutrition [16].

Normalization of weight-for-age of involved patients made it possible to directly compare nutrition status for patients at different ages. All analyses were performed using IBM SPSS Statistics 22 (IBM, Armonk, NY, USA). Descriptive data were presented as the mean ± standard deviation (SD). Paired t-test was used for comparing Z-scores of preoperative and latest follow-ups. The Pearson correlation coefficient (*r*) was used to test bivariate relationships. *p*-values ˂ 0.05 were considered to indicate statistical significance.

The study protocol was approved by board of ethical committee of Peking Union Medical College Hospital and was performed in accordance with the relevant guidelines.

## Results

In total of 52 patient were involved with 34 (71%) females. The mean age at initial growing-rod implantation was 6.9 years. The minimal follow-up period was 36 months, and the median follow-up time was 72 months. At the time of the initial surgery, the average body weight of involved patients was 19.9 kg and the average Z-score was − 0.94, meaning that the average body weight of these EOS patients was 0.94 standard deviations below the average body weight of the age-matched general population in China (Table 1). The average pre-operative Cobb angle was 72.3°. The diagnosis of involved patients includes: 33 (63.5%) congenital scoliosis, 9 (17.3%) syndromic scoliosis, 2 (4%) idiopathic scoliosis, and 8 neuromuscular scoliosis, including 6 patients with syringomyelia and 2 patients with split cord malformations in thoracic segments. Syndromic scoliosis includes: neurofibromatosis [6], Freeman–Sheldon syndrome [1], Marfanoid syndrome [1] and Jarcho–Levin syndrome [1].

**Table 1:** Patient characteristics

	Mean ± SD	Range
Age (year) (N = 52, 100%)	6.9 ± 2.5	2–10
Male (N = 18, 34.6%)		
Female (N = 34, 65.4%)		
Height (cm)	110.0 ± 16.1	79–151
Weight (kg)	19.9 ± 6.6	10–47
Z-score	− 0.94 ± 1.30	− 3.01–2.14

*N* numbers, *SD* standard deviation

**Fig. 1:**
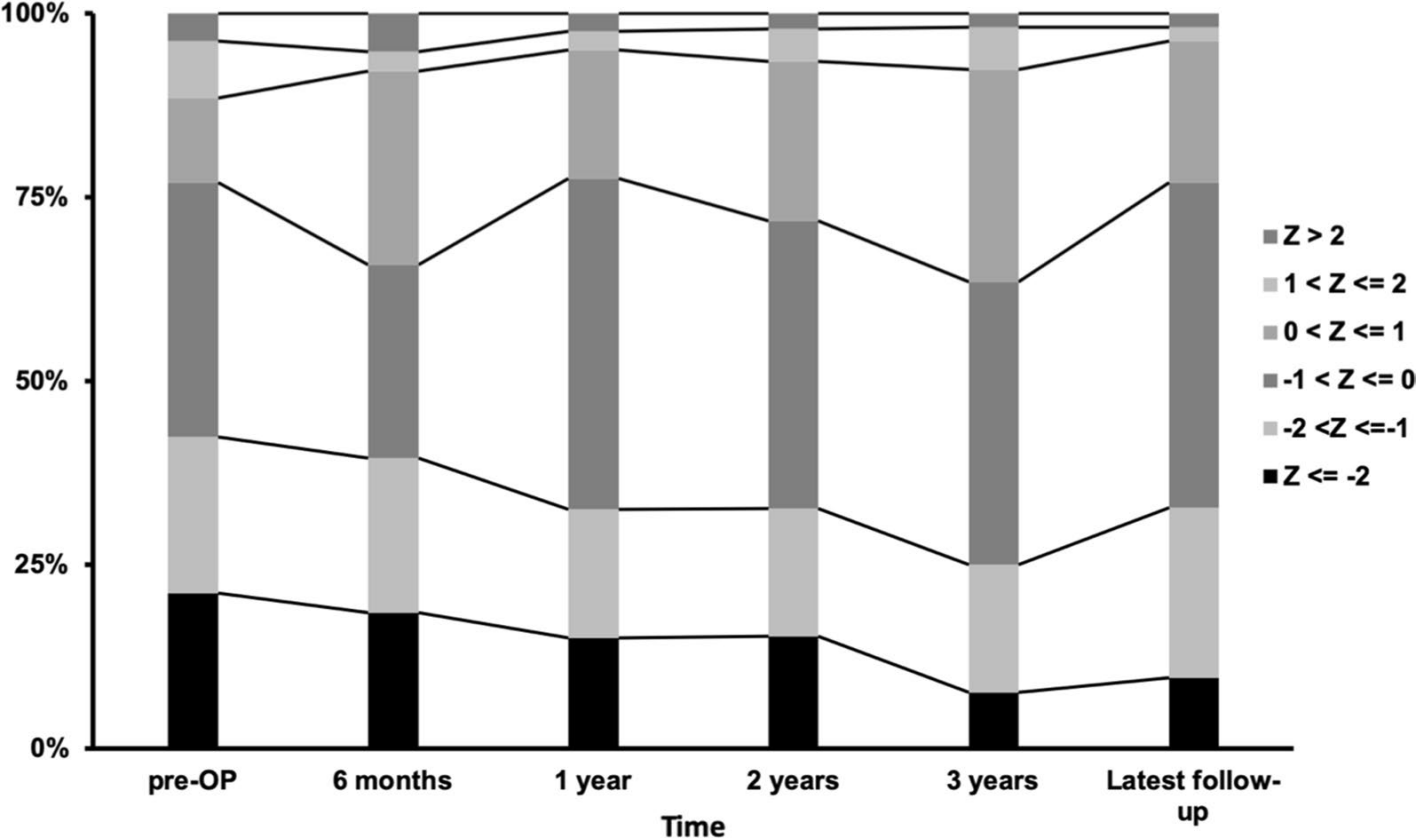
Percentile of patients with different body weight group in each postoperative time. Each column indicates 100% patients of each time point. Black portions indicate the percentile of patients with Z-score below − 2, who were diagnosed of malnutrition

**Fig. 2:**
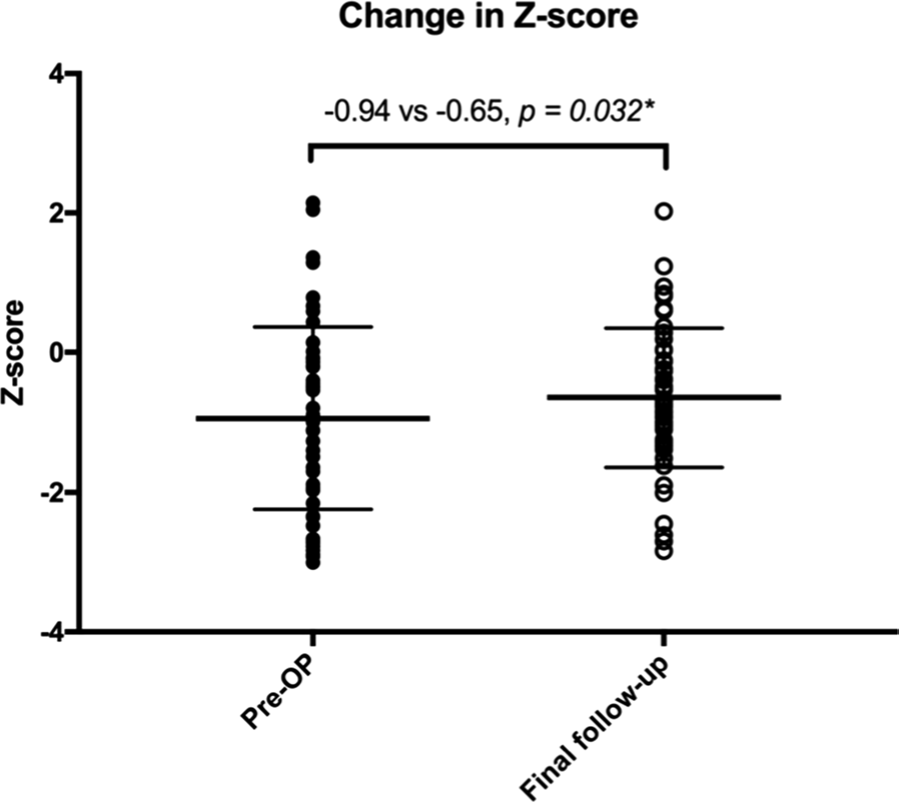
Change in Z-scores after growing-rod implantation and lengthenings. Each dot indicates a preoperative Z-score, each circle indicates Z-score at the final follow-up. The lines indicate mean and ± 1 SD. *p* < 0.05 is indicated by *; p < 0.01 is indicated by **

Preoperatively, the Z-score of 15 (21.15%) patients was below − 2, meaning the incidence of malnutrition was 15% in the enrolled patients. Postoperatively, the proportion of patients with Z-score below − 2 was 21.2% at 6 months, 18.4% at 1 year, 15.2% at 2 years and 9.6% at the latest follow-up (Fig. 1). The average Z-score at the latest follow-up was − 0.65, which was significantly increased compared to the preoperative Z-score (− 0.65 vs − 0.94, *p* < 0.05, Fig. 2).

**Fig. 3:**
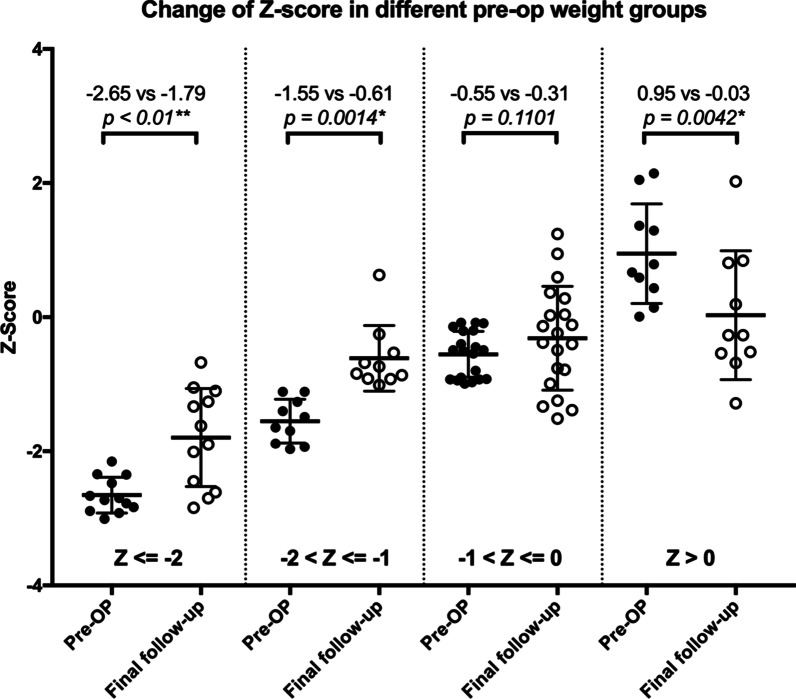
Comparison of change of Z-score between preoperative and final-follow-up in different preoperative Z-score groups. Each dot indicates a preoperative Z-score, each circle indicates Z-score at the final follow-up. The lines indicate mean and ± 1 SD. *p* < 0.05 is indicated by *; p < 0.01 is indicated by **

**Fig. 4:**
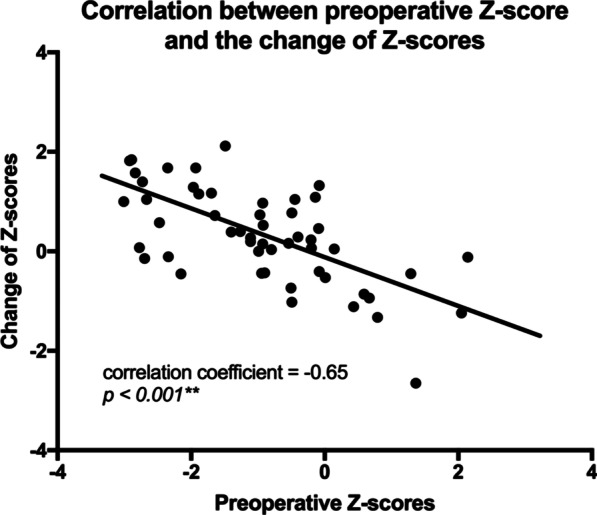
Correlation between preoperative Z-score and change in Z-score. X-axis indicates pre-operative Z-score and Y-axis indicates the difference of Z-score between preoperatively and the final follow-up. Each dot indicates a case*. p* < 0.01 is indicated by **

A significant correlation was found between the Z-score before the initial surgery and the change of Z-scores. The Z-score increase is more significant in patients with preoperative Z-score below − 1. For patients with preoperative Z-scores below − 2, their average Z-score increased from − 2.65 preoperatively to − 1.79 at the latest follow-up, and for patients with preoperative Z-scores between − 1 and − 2, their average Z-score increased from − 1.55 preoperatively to − 0.61 at the latest follow-up, respectively (*p* < 0.01). Combining two groups together, Z-score increased significantly from in average − 2.15 preoperatively to − 1.26 at the final follow-up for patients with preoperative Z-score below − 1 (*p* < 0.001). But for patient with preoperative Z-score between − 1 and 0, their Z-score gain was not significant. And for patients with preoperative Z-score above 0, their average Z-score even decreased from 0.95 to 0.03 (*p* < 0.01). For all patient with preoperative Z-score above − 1, the change in Z-score was not significant (*p* = 0.36) (Table 2 and Fig. 3). There was a significant negative correlation between change in Z-score and preoperative Z-score, with correlation coefficient − 0.65, *p* < 0.001 (Fig. 4). This correlation was not found between change in Z-score and age at initial surgery (*p* = 0.43).

**Table 2:** Change of Z-score in different pre-operative weight groups

Group	N	M pre-op Z	M final F/U Z	*p*
Z ≦ − 2	12	− 2.65	− 1.79	0.0022**
− 2 < Z ≦ − 1	20	− 1.55	− 0.61	0.0003**
− 1 < Z ≦ 0	10	− 0.55	− 0.31	0.2965
Z > 0	10	0.95	0.03	0.0036**
All	52	− 0.94	− 0.65	0.0322*

Patient weights were grouped by pre-operative Z-scores

*N* case number, *M* mean, *F/U* follow-up

p < 0.05 is indicated by *; p < 0.01 is indicated by **

Z-scores of patients with all diagnosis increased at the last follow-up. This is significant in congenital scoliosis and neuromuscular scoliosis patients (p < 0.05). While it is not significant for patients with syndromic scoliosis (Table 3).

**Table 3:** Change in Z-score in different diagnosis

Group	N	Preoperative	Last follow-up	*p*
Congenital	33	− 0.93	− 0.56	0.0253*
Syndromic	9	− 0.81	− 0.75	0.8608
Neuromuscular	8	− 1.44	− 0.79	0.0261*

*N* case numbers

p < 0.05 is indicated by *; p < 0.01 is indicated by **

## Discussion

Our study revealed that growing-rod implantation and lengthening procedures can improve weight-for-age position of EOS patients in the 3-year follow-up period. The change in body weight Z-score was more significant in patients with preoperative Z-score lower than − 1. The preoperative Z-score had negative correlation with the increase of Z-score during the follow-up. The nutrition status of enrolled patients, spatially underweight patients, benefited from growing-rod implantation in this study.

According WHO Database on Child Growth and Malnutrition, Z-score lower than − 2, or the lowest 2.3% weight-for-age in general population, is commonly used as the cut-off for malnutrition [16]. Malnourished children have increased risk for infection, fractures, surgical complications [17–20] or even early deaths [21]. In this study, the prevalence of malnutrition before growing-rod implantation was 28.9%, and was reduced to 7.7% at the final follow-up. Therefore, for children with EOS, growing-rod surgery is likely to reduce their future risk of increased mortality and morbidity [22].

Myung et al. studied 88 EOS patients treated with growing rods. They found significant increase in mean postoperative weight percentile, however, no correlation between weight at initial surgery and percentile gain was found [12]. Harris et al. studied 287 EOS patients treated with VEPTR or growing-rods and found that both techniques increased weight percentile of patients. Previous studies observed only weight percentile changes, however, percentile data only indicate a rough range. In this study, we used large-scale census-based data to normalize weight-for-age into Gaussian distribution replacing percentile to Z-score, which indicates of the relative weight of children in age-matched population more statistical effectively and is more commonly adopted in nutritional studies [23]. Our study also found a strong relationship between the gain in Z-score and preoperative Z-score, meaning severely malnourished patients benefit more from growing-rod surgeries.

The reference curve we applied in calculation was based on Chinese population census, so it gives most accurate position of the weight-for-age among Chinese children. If applying the same reference curve to other nationalities, the result may not as accurate as in Chinese population.

In recent years, new growing-rod techniques that do not required repeated lengthening surgeries or repeated anesthesia have emerged. Miladi reported an external maneuver-controlled growing device that can be applied on the ribs, vertebra and pelvis of EOS patients [24]. Magnetic-controlled growing rods also showed clinical significance [25].

This study has three main limitations: (1) the patient number involved in this study was limited; (2) lack of longer follow-up until the final fusion surgery after a series of lengthening procedures; (3) lacking a control group without surgical interventions.

## Conclusions

The growing rod implantation and lengthening procedures significantly improves the nutrition status of patients with early-onset scoliosis. The gain of body weight is more significant in patients with lower preoperative body weight.

## Data Availability

The datasets used and/or analysed during the current study available from the corresponding author on reasonable request.
